# Long non-coding RNA-CRNDE: a novel regulator of tumor growth and angiogenesis in hepatoblastoma

**DOI:** 10.18632/oncotarget.14992

**Published:** 2017-02-02

**Authors:** Rui Dong, Xiang-Qi Liu, Bin-Bin Zhang, Bai-Hui Liu, Shan Zheng, Kui-Ran Dong

**Affiliations:** ^1^ Department of Pediatric Hepatobiliary Surgery, Children's Hospital of Fudan University, Key Laboratory of Neonatal Disease, Ministry of Health, Shanghai 201102, China

**Keywords:** long non-coding RNA, tumor angiogenesis, tumor growth, mTOR signaling

## Abstract

Long non-coding RNAs (lncRNAs) are involved in many biological processes, such as angiogenesis, invasion, cell proliferation, and apoptosis. They have emerged as key players in the pathology of several tumors, including hepatoblastoma. In this study, we elucidate the biological and clinical significance of CRNDE up-regulation in hepatoblastoma. CRNDE is significantly up-regulated in human hepatoblastoma specimens and metastatic hepatoblastoma cell lines. CRNDE knockdown reduces tumor growth and tumor angiogenesis *in vivo*, and decreases hepatoblastoma cell viability, proliferation, and angiogenic effect *in vitro*. Mechanistic studies show that CRNDE knockdown plays its anti-proliferation and anti-angiogenesis role via regulating mammalian target of rapamycin (mTOR) signaling. Taken together, this study reveals a crucial role of CRNDE in the pathology of hepatoblastoma. CRNDE may serve as a promising diagnostic marker and therapeutic target for hepatoblastoma.

## INTRODUCTION

Hepatoblastoma is one of the most common liver tumors in children, accounting for more than 65% of childhood liver malignancies [1, 2]. Hepatoblastoma is different from adult liver cancers. It is not associated with hepatitis virus infection, cirrhosis, or other underlying liver pathology [1, 3]. The survival rate of hepatoblastoma is very high if hepatoblastoma was completely resected. However, the prognosis of unresectable metastatic cases is still very poor [4, 5]. Thus, it is required to understand the mechanism of hepatoblastomagenesis and develop effective diagnostic biomarkers as well as novel therapeutic targets for the patients with hepatoblastoma.

Angiogenesis is a common feature in nearly all tumors and associated with tumor grade and malignancy [6, 7]. Angiogenesis provides enough blood supply to meet the requirement of rapid tumor growth. Angiogenesis helps tumor cells to spread with the blood stream to distant organs [8]. Angiogenesis also plays a crucial role in the development and metastasis of hepatoblastoma [9]. However, the mechanism orchestrating vascularization is still unclear in hepatoblastoma.

Long non-coding RNAs (lncRNAs) are non-coding transcripts more than 200 nucleotides in length. They are ubiquitously expressed in mammalian genomes, and participate in biological processes, such as chromatin remodeling, transcription regulation, epigenetic regulation, and mRNA processing [10, 11]. LncRNAs are dysregulated in a wide range of human cancers, such as prostate cancer, gastric cancer, breast cancer, and hepatocellular carcinoma (HCC) [12–15]. We have performed genome-wide lncRNA analysis to identify hepatoblastoma-related lncRNAs. LncRNA-CRNDE is shown to be differentially expressed between hepatoblastoma and normal liver tissue [16]. However, the role and mechanism of CRNDE in tumor growth and angiogenesis in hepatoblastoma is still unclear. In this study, we elucidate the biological and clinical significance of CRNDE up-regulation in hepatoblastoma. We show that CRNDE knockdown inhibits tumor angiogenesis and tumor growth via activating mammalian target of rapamycin (mTOR) signaling.

## RESULTS

### CRNDE expression is significantly up-regulated in hepatoblastoma specimens and cell lines

**Figure 1:**
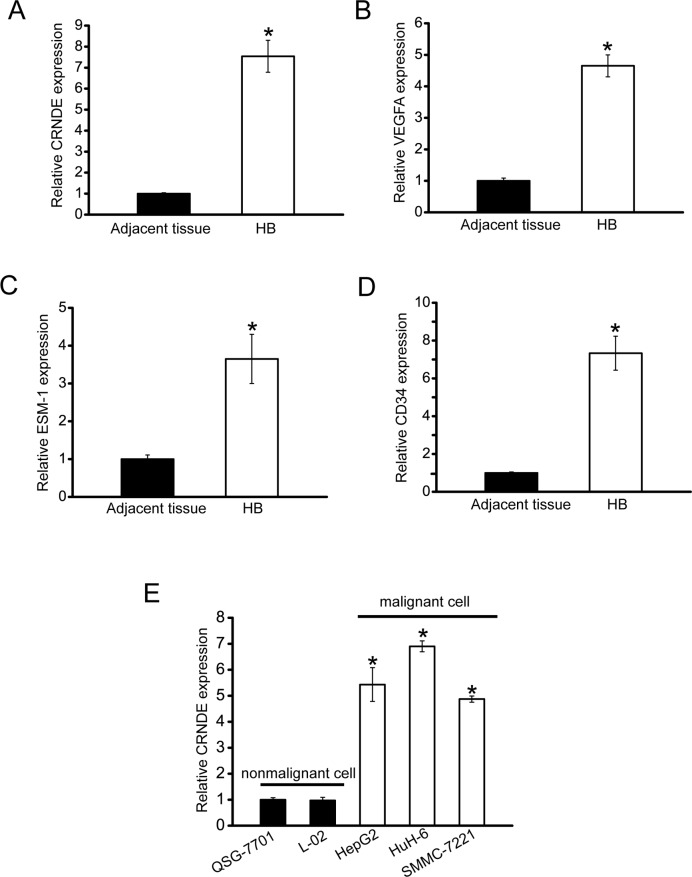
CRNDE is significantly up-regulated in human hepatoblastoma specimens and cell lines **A**. Total RNAs were extracted from 8 human hepatoblastoma specimens (HB) and 8 matched adjacent non-cancerous tissues. qRT-PCRs were conducted to detect CRNDE expression. All data were from three independent experiments (Mann-Whitney *U*-test; *P*=0.0008; *P<0.05 versus adjacent tissue). **B-D**. VEGFA, ESM-1, and CD34 levels in human hepatoblastoma specimens and matched non-cancerous tissues were detected by ELISA assays (n=8). All data were analyzed by Mann-Whitney *U*-test [*P*=0.0043 (B); *P*=0.0136 (C); *P*=0.0004 (D); *P<0.05 versus adjacent tissue]. **E**. qRT-PCRs were conducted to detect CRNDE expression in nonmalignant QSG-7701 and L02 hepatocytes, and malignant SMMC-7221, HuH-6, and HepG2 cells (n=4). CRNDE expression in QSG-7701 cells was taken as value 1. All data were expressed as relative change compared with CRNDE expression in QSG-7701 cells. Statistical difference was analyzed by Student's *t*-test (two-sided). *P*=0.612 (L02); *P*=0.0021 (HepG2); *P*=0.0005 (HuH-6); *P*=0.0028 (SMMC-7221). *P<0.05 versus CRNDE expression in QSG-7701 cells.

To determine whether CRNDE is involved in tumorigenesis, we collected hepatoblastoma specimens to detect CRNDE expression pattern. CRNDE expression was significantly up-regulated in hepatoblastoma tissues compared with the matched normal tissues (Figure 1A). Meanwhile, we estimated tumor angiogenesis by detecting VEGFA, ESM-1 (endothelial cell specific molecule 1), and CD34 levels by ELISA assays. The expression of VEGF, ESM-1, and CD34 was positively associated with CRNDE levels in the hepatoblastoma tissues (Figure 1B–1D). Increased CRNDE expression was also observed in all examined hepatocellular carcinoma and hepatoblastoma cells compared with the nonmalignant QSG-7701 and L01 hepatocytes (Figure 1E). Taken together, these results suggest that CRNDE is a potential regulator of hepatoblastoma development and tumor angiogenesis.

### CRNDE knockdown inhibits tumor growth and tumor angiogenesis *in vivo*

**Figure 2:**
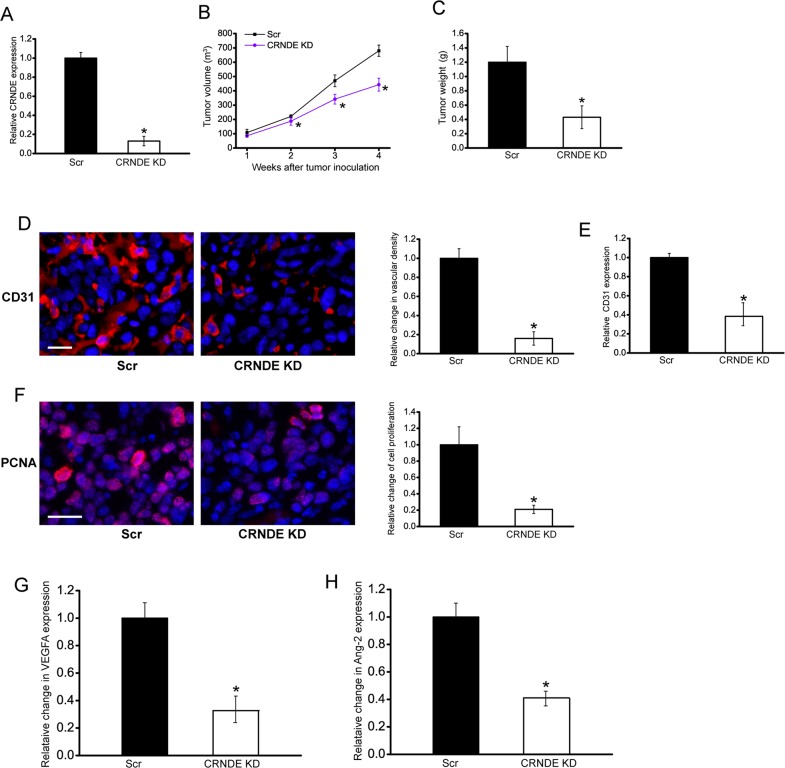
CRNDE knockdown inhibits tumor growth and tumor angiogenesis *in vivo* An *in vivo* hepatoblastoma model was established by injection of CRNDE knockdown HuH-6 cells and scrambled shRNA-transfected HuH-6 cells into the left flank of nude mice (C57BL/6). CRNDE expression in the tumors isolated from CRNDE knockdown (KD) group and negative control group (Scr) were detected by qRT-PCRs after 4-week tumor inoculation (**A**; Student's *t*-test; *P*=0.0023). The volume of solid tumor was measured every week using a vernier caliper [**B**; n=6; Mann-Whitney *U*-test; *P*=0.0285 (2 w), *P*=0.0209 (3 w), *P*=0.0134 (4 w)]. The tumors were weighed immediately after 4-week tumor inoculation (**C**; Mann-Whitney *U*-test; *P*=0.0011). CD31 and PCNA staining was performed to detect microvascular density **D**. and cell proliferation **F**. in the hepatoblastoma tissues after 4-week tumor inoculation. Vessels with a clearly defined lumen or well-defined linear vessel shape but not single endothelial cells were considered for microvessel density assessment. A representative image and statistical result was shown [*n*=10 slices; Mann-Whitney *U*-test; *P*=0.0034 (D) and *P*=0.0022 (F)]. Scale bar: 100 μm. ELISA assays were conducted to detect CD31 expression after 4-week tumor inoculation (**E**, *n*=5; Mann-Whitney *U*-test; *P*=0.0024). ELISAs were conducted to compare VEGFA **G**. and Ang-2 **H**. levels between hepatoblastoma tissue of CRNDE KD group and Scr group after 4-week tumor inoculation (*n*=6; Mann-Whitney *U*-test; *P*=0.0124). “*” indicated significant difference between Scr group and CRNDE KD group.

To explore the function significance of CRNDE induction, we employed hepatoblastoma xenograft mouse model to determine the effect of CRNDE knockdown on tumor growth and angiogenesis. CRNDE knockdown HuH-6 cells or scrambled shRNA-transfected HuH-6 cells were subcutaneously injected into the left flank of nude mice (C57BL/6). CRNDE expression was obviously decreased in CRNDE knockdown group (Figure 2A). Moreover, the tumor volume in CRNDE knockdown group was significantly smaller than that in scrambled shRNA-transfected group after 2-week hepatoblastoma inoculation (Figure 2B), which was consistent with tumor weight reduction (Figure 2C).

We also employed CD31 staining to detect tumor vascular change. Compared with the control group, subcutaneous xenograft tumors in CRNDE knockdown group had lower microvessel density (Figure 2D). ELISA assays showed that CRNDE knockdown group had lower CD31 expression levels (Figure 2E). PCNA staining showed that CRNDE knockdown obviously reduced the proportion of proliferating (PCNA^+^) tumor cells (Figure 2F). VEGFA and Ang-2 are two important regulators of tumor angiogenesis. ELISA assays showed that CRNDE knockdown significantly decreased VEGFA and Ang-2 levels in the hepatoblastoma tissue (Figure 2G and 2H). These results suggest that CRNDE knockdown may attenuate tumorigenesis through anti-proliferation and anti-angiogenesis during hepatoblastoma development.

### CRNDE knockdown decreases cell viability, proliferation, and tube formation *in vitro*

**Figure 3:**
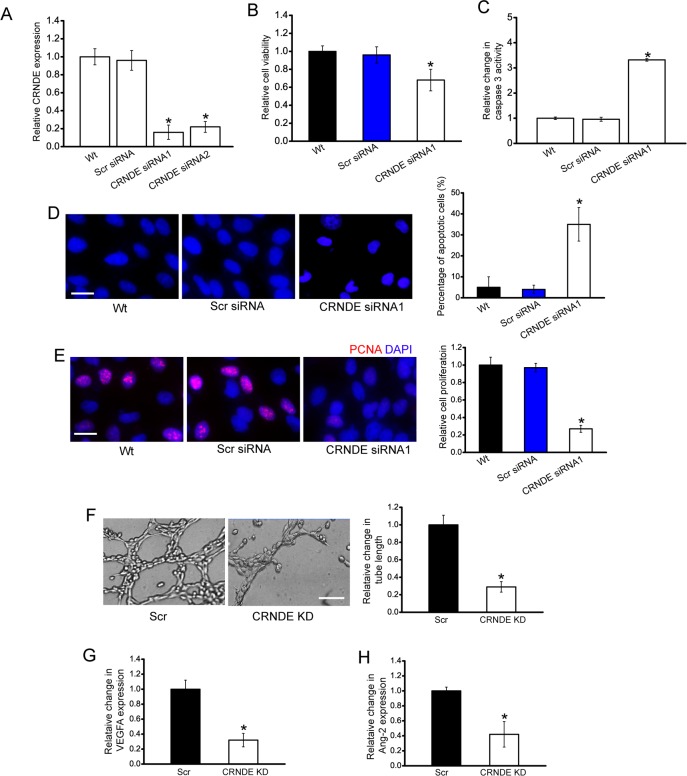
CRNDE knockdown decreases cell viability, proliferation, and tube formation *in vitro* **A**. HuH-6 cells were transfected with scrambled siRNA (Scr), CRNDE siRNA1, CRNDE siRNA2, or left untreated (Wt) for 48 h. qRT-PCRs were conducted to detect CRNDE expression. The data were shown as fold increase compared with Wt group. “*” indicated significant difference compared with Wt group (*n*=4; Student's *t*-test; *P*=0.0146). **B-E**. HuH-6 cells were transfected with Scr siRNA, CRNDE siRNA1, or left untreated (Wt) for 48 h. Cell viability was detected using CCK-8 method (**B**; *n*=4; Student's *t*-test; *P*=0.0209). Caspase 3 activity (**C**; *n*=4; Student's *t*-test; *P*=0.0112) and Hoechst staining was conducted to detect cell apoptosis (**D**; *n*=4; Student's *t*-test; *P*=0.0145; Scale bar: 10 μm). PCNA immunofluorescence staining and quantitative analysis was conducted to detect cell proliferation (**E**; *n*=4; Student's *t*-test; *P*=0.0119; Scale bar, 10 μm). **F**. HUVECs were cultured in 24-well plates coated with matrigel in TCM derived from HuH-6 cells transfected with CRNDE siRNA1, or Scr siRNA, or without any transfection, and added to the matrigel-coated wells. A representative image of tube formation and the length of tube formation was shown (n=4; Student's *t*-test; *P*=0.0143). Scale bar: 50 μm. **G, H**. The secretion of VEGFA and Ang-2 in CRNDE siRNA or scrambled siRNA-transfected HuH-6 cells was detected using ELISA assays [n=4; Student's *t*-test; *P*=0.0106 (G), *P*=0.0187 **(H)**].

We designed two different CRNDE siRNAs, and revealed that CRNDE siRNA transfection significantly reduced CRNDE expression in HuH-6 cells (Figure 3A). CRNDE siRNA1 transfection displayed a significant suppressive effect on cell viability (Figure 3B). Hoechst staining and caspase 3 activity assay revealed that more apoptotic cells were induced upon CRNDE siRNA1 transfection in HuH-6 cells (Figure 3C). PCNA staining showed that CRNDE knockdown reduced the proliferation of HuH-6 cells (Figure 3D). Moreover, we showed that CRNDE siRNA2 transfection had a similar effect on HuH-6 cell function as CRNDE siRNA1 transfection (Supplementary Figure 1). We also used HepG2 hepatoblastoma cell line to investigate the functional significance of CRNDE expression alteration *in vitro*. CRNDE knockdown significantly reduced the viability and proliferation, and accelerated the development of HepG2 cell apoptosis (Supplementary Figure 2).

The angiogenic effect of CRNDE was also estimated. HuH-6 cells were transfected with CRNDE siRNA and scrambled siRNA. The conditional medium was collected, and then incubated with HUVECs. A dramatically suppressive effect of tube formation was observed in HUVEC cells treated with CRNDE knockdown condition medium (Figure 3F). ELISA assays indicated that the secretion of VEGFA and Ang-2 was reduced by CRNDE knockdown in HuH-6 cells (Figure 3G and 3H).

### CRNDE regulates the development of hepatoblastoma through mTOR signaling

**Figure 4:**
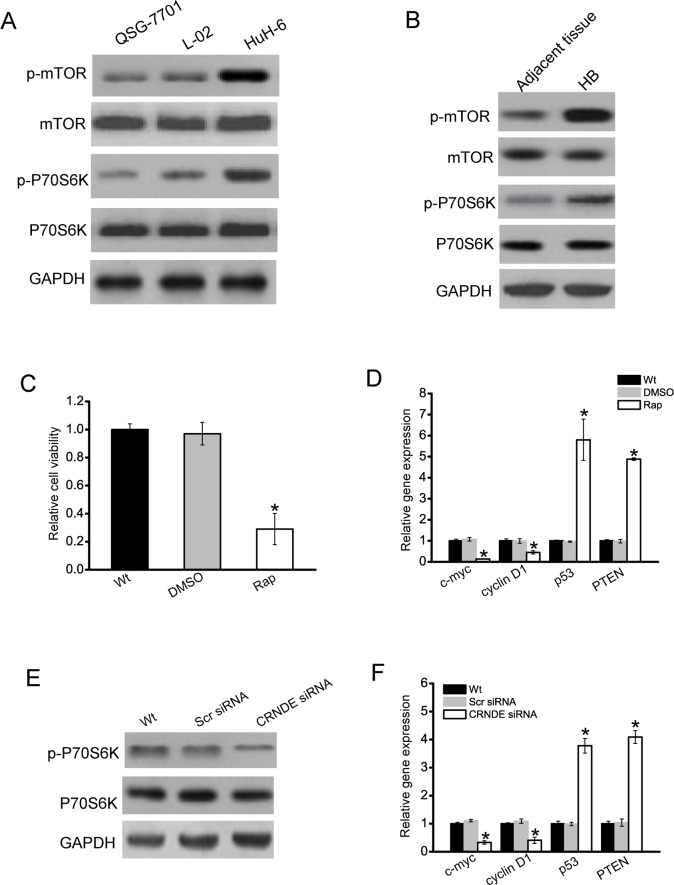
CRNDE regulates the development of hepatoblastoma through mTOR signaling **A**. Total protein was extracted from QSG-7701, L02 hepatocytes, and HuH-6 cells (n=4). **B**. Total protein was extracted from 5 human hepatoblastoma specimens (HB) and 5 matched adjacent non-cancerous tissues. Western blots were conducted to detect the expression of mTOR, p-mTOR, P70S6K, and p-P70S6K. GAPDH was detected as the internal control. A representative image was shown. **C**. HuH-6 cells were treated with rapamycin (Rap), DMSO, or left untreated (Wt). Cell viability was detected using CCK-8 method (*n*=4; Student's *t*-test; *P*=0.0121). **D**. HuH-6 cells were treated with rapamycin (Rap), DMSO, or left untreated (Wt). Relative expression of c-myc, cyclinD1, p53, and PTEN was detected by qRT-PCRs (*n*=4; Student's *t*-test; *P*=0.0143, *P*=0.0209, *P*=0.0047, and *P*=0.0099, respectively). **E**. HuH-6 cells were transfected with scrambled siRNA (Scr), CRNDE siRNA, or left untreated (Wt) for 48 h. Western blots were conducted to detect the expression of P70S6K and p-P70S6K. GAPDH was detected as the internal control. A representative image was shown. **F**. HuH-6 cells were transfected with scrambled siRNA (Scr), CRNDE siRNA, or left untreated (Wt) for 48 h. Relative expression of c-myc, cyclinD1, p53, and PTEN was detected by qRT-PCRs (*n*=4; Student's *t*-test; *P*=0.0198, *P*=0.0211, *P*=0.0078, and *P*=0.0107, respectively).

During cancer cell metastasis, mTOR signaling pathway plays a critical role in cancer cell metastasis, and is aberrantly regulated [17, 18]. We then determined whether mTOR signaling is activated in hepatoblastoma cells. Phosphorylated mTOR and P70S6K (a serine/threonine kinase that is directly downstream of mTOR) expression was much higher in hepatoblastoma cells (HuH-6) than that in nonmalignant QSG-7701 and L02 cells, indicating that mTOR signaling was activated in hepatoblastoma cells (Figure 4A). Moreover, phosphorylated mTOR and P70S6K level was significantly higher in hepatoblastoma tissues compared with the matched normal tissues (Figure 4B). Once mTOR signaling was significantly blocked with rapamycin, a specific inhibitor targeting mTOR, the rate of HuH-6 cell proliferation was decreased (Figure 4C). The expressions of cell proliferation-related genes, such as c-myc and cyclin D1, were obviously down-regulated in HuH-6 cells treated with rapamycin, while tumor suppressor genes, like p53 and PTEN, were obviously up-regulated (Figure 4D).

We further investigated whether CRNDE regulates HuH-6 cell proliferation through mTOR signaling. We showed that phosphorylation level of P70S6K was significantly reduced in CRNDE knockdown cells (Figure 4E). CRNDE knockdown significantly decreased the expression of c-myc and cylcin D1, but increased the expression of p53 and PTEN (Figure 4F).

### CRNDE-mTOR signaling regulatory network is critical for hepatoblastoma cell function and angiogenic effect

**Figure 5:**
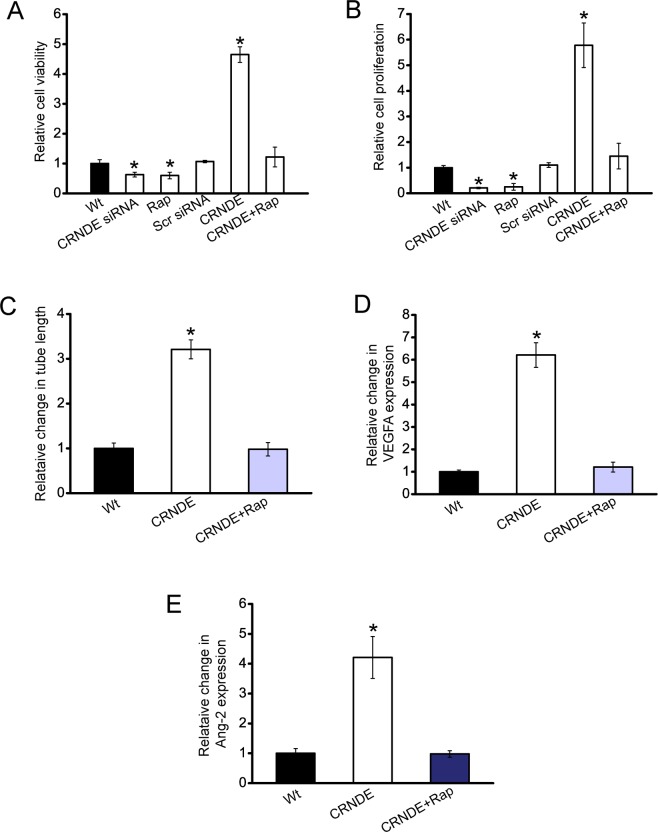
CRNDE-mTOR signaling regulatory network is involved in regulating hepatoblastoma cell function and angiogenic effect **A, B**. HuH-6 cells were transfected with scrambled siRNA (Scr), CRNDE siRNA, left untreated (Wt), overexpressed CNDE with or without rapamycin treatment. Cell viability was detected using CCK-8 method (**A**; *n*=4; Student's *t*-test; *P*=0.0135, 0.0151, 0.0032, respectively). PCNA staining and quantitative analysis was conducted to detect cell proliferation (**B**; *n*=4; Student's *t*-test; *P*=0.0165, 0.0176, 0.0011, respectively). **C-E**. HUVECs were cultured in 24-well plates coated with matrigel in TCM derived from HuH-6 cells transfected with CRNDE, or CRNDE plus rapamycin treatment, or without any transfection, and added to matrigel-coated wells. Quantitative analysis of tube formation were shown (**C**; n=4; Student's *t*-test; *P*=0.0133). The secretion of VEGFA (**D**; n=4; Student's *t*-test; *P*=0.0087) and Ang-2 (**E**; n=4; Student's *t*-test; *P*=0.005) in HuH-6 cells was detected using ELISA assays.

We then investigated whether CRNDE-mTOR signaling regulatory network is involved in regulating HuH-6 cell function and angiogenic effect. CCK-8 assay and PCNA staining showed that cell viability and proliferation was significantly decreased in CRNDE knockdown cells, similar to rapamycin-treated cells. Rapamycin treatment could inhibit CRNDE overexpression-induced up-regulation of cell proliferation and cell viability (Figure 5A and 5B).

CRNDE was then overexpressed in HuH-6 cells, and treated with or without rapamycin. The conditional medium was then collected, and incubated with HUVECs. CRNDE-overexpressed medium significantly increased the tube formation ability, whereas rapamycin treatment exerted suppressive effect on tube formation of HUVECs (Figure 5C). Moreover, ELISA assays revealed that the secretion of VEGFA and Ang-2 was significantly increased upon CRNDE overexpression, which was reduced by rapamycin treatment (Figure 5D and 5E). Collectively, these results reveal a direct correlation between CRNDE expression and mTOR signaling activation.

## DISCUSSION

Hepatoblastoma is an uncommon liver malignancy in infants and children. Inhibition of tumor growth and angiogenesis contributes to the development of efficient treatment [16, 19]. In this study, we show that CRNDE knockdown inhibits tumor growth and tumor angiogenesis *in vivo*, and reduces hepatoblastoma cell viability and angiogenic effect *in vitro*. CRNDE up-regulation contributes to unusual hypervascularity of hepatoblastoma.

Mounting evidence has shown that modification of lncRNA expression is tightly associated with tumor formation. We have identified 2736 differentially expressed lncRNAs between hepatoblastoma and normal liver tissue [16]. We also show that TUG1 is a promising therapeutic target for aggressive, recurrent, or metastatic hepatoblastoma [9]. Here we put the spotlight on the function of CRNDE, a lncRNA up-regulated in hepatoblastoma. We show that that CRNDE is involved in hepatoblastoma through regulating tumor growth and tumor angiogenesis.

CRNDE is the gene symbol for Colorectal Neoplasia Differentially Expressed (non-protein-coding), a long non-coding RNA. CRNDE plays important role in development. CRNDE expression appears highest at the early stage of human development and progressively decreases thereafter. It is involved in regulating multipotency, differentiation, and gametogenesis. CRNDE expression is significantly up-regulated in a number of neoplastic diseases, such as colorectal cancer and glioma [20, 21]. CRNDE is identified as an oncogene for its function in promoting cell growth and migration. Increased CRNDE expression during neoplasia may be a response to perturbations in upstream signaling pathways, for example the MAP kinase pathway. CRNDE acts through epigenetic mechanisms to regulate cell function, which may relate to its deregulation in cancer, such as histone acetylation or methylation [22–24]. We speculated that CRNDE is also shown as an oncogene in hepatoblastoma. CRNDE is up-regulated during the development of hepatoblastoma. Its knockdown inhibits tumor growth angiogenesis *in vivo* and reduces hepatoblastoma cell viability *in vitro*. CRNDE up-regulation contributes to the development of hepatoblastoma.

The mTOR signaling pathway plays an important role in cell growth and proliferation in response to mitogen, nutrient, and energy status [25, 26]. Aberrant activation of mTOR pathway either by loss of tumor suppressors or activation of oncogenes promotes tumor growth, which has been shown in many malignant cell lines [24, 27]. We reveal a direct interaction between CRNDE and mTOR in hepatoblastoma. mTOR signaling pathway is activated in hepatoblastoma cells and hepatoblastoma tissues. CRNDE knockdown significantly inhibits the activation of mTOR signaling. Moreover, inhibition of mTOR signaling could inhibit CRNDE overexpression-induced up-regulation of hepatoblastoma cell viability and abnormal angiogenic effect. Thus, it not surprised that CRNDE could play a pro-oncogenic role through the modulation of mTOR signaling.

In conclusion, we show that CRNDE is significantly up-regulated in hepatoblastoma samples and metastatic hepatoblastoma cell lines. CRNDE up-regulation contributes to tumor growth and hypervascularity of hepatoblastoma via mTOR signaling activation. CRNDE plays an important role in the development of hepatoblastoma and finding out the underlying mechanism could be a potential novel strategy for the treatment of hepatoblastoma.

## MATERIALS AND METHODS

### Human tissue specimens and cell lines

Human hepatoblastoma and adjacent non-tumor liver tissues were collected from the patients undergoing resection of hepatoblastoma in Children hospital, Fudan University, China. Informed consent was obtained from every patient. This study was also approved by the Institute Research Ethics Committee. Tumor cell lines, HepG2, HuH-6, and SMMC-7221, and nonmalignant QSG-7701 and L01 hepatocytes were cultured in Dulbecco's Modified Eagle's medium supplemented with 10% fetal bovine serum (FBS) in a humidified incubator with 5% CO_2_ at 37°C.

### Small interfering RNA transfection

CRNDE small interfering RNA (siRNA) was purchased from Qiagen (Hilden, Germany). The target sequence was 5′-GTGCTCGAGTGGTTTAAAT-3′ and 5′-GTCTGCAATTCATAATGGA-3′. The negative control siRNA was also purchased from Qiagen. The cells were incubated with either CRNDE siRNA or negative control siRNA using LipoMax Transfection reagent according to the protocol.

### Quantitative reverse transcription-PCR (qRT-PCR)

Total RNAs were isolated from cells and tissues using TRIZOL reagent (Life technologies) according to the manufacturer's instructions. About 2 μg of total RNA was subjected for reverse transcription using the M-MLV reverse transcriptase (Promega). qRT-PCRs were conducted using an ABI 7300 real-time RT-PCR system with SYBR® Green Real-time PCR Master Mix (Takara). Gene expression was normalized to GAPDH expression level. PCR products were verified by melting curve analysis. Data analysis was performed using the 2^−ΔΔCt^ method [28]. The primer sequences were as follows: CRNDE primers, forward: 5′- CGATCGCGCTATTGTCATGG-3′, reverse: 5′-TCCGCC TCGCTTAGACATTG-3′; glyceraldehyde 3-phosphate dehydrogenase (GAPDH) primers forward: 5′-CGCTCT CTGCTCCTCCTGTTC-3′, GAPDH reverse: 5′-ATCC GTTGACTCCGACCTTCAC-3′.

### Tube formation assay

HUVECs were seeded into a 96-well culture plate pre-coated with Matrigel (BD Biosciences) with or without the medium containing 5% FBS and 1% penicillin/streptomycin or CM of HuH-6 cells. After 24-h incubation, tube formation was observed using a phase contrast microscopy, and quantified by calculating tube length.

### Western blotting

Cell lysates were subjected to sodium dodecyl sulfate-polyacrylamide gel electrophoresis (SDS-PAGE), transferred to the nitrocellulose membrane, immunoblotted with the primary antibody, and then immunoblotted with the secondary antibody at room temperature. Protein signaling was visualized by the enhanced chemiluminescence kit (Pierce). GAPDH was detected as a loading control.

### TCM preparation

Tumor cells (1 × 10^5^) were transfected with the required RNA oligonucleotides in a 6-well plate. The medium was removed 36 h after transfection. Cells were washed with phosphate-buffered saline (PBS), and then cultured for additional 12 h. The TCM was collected, centrifuged at 500 × *g* to remove these detached cells, and then centrifuged at 12, 000 × *g* to discard cell debris. TCM was then stored in aliquots at -80°C [9].

### Cell viability assay

Cell viability was detected using the CCK-8 kit (Dojindo Laboratories, Kumamoto, Japan) according to the manufacturer's instruction [12]. Cells were cultured in a 96-well plate at a concentration of 1 × 10^4^ cells/ml. OD450 was detected after 3-h incubation with CCK-8.

### Cell apoptosis assay

Cell apoptosis was detected using Hoechst 33342 staining [29]. After the required treatment, cells were stained with Hoechst 33342 (5 μg/ml, Sigma) for 10 min. The changes in nuclear morphology were detected by fluorescence microscopy using a filter for Hoechst 33342 (365 nm). The fractions of cells (at least 1 × 10^3^ per condition) displaying pycnotic nuclei were scored under fluorescence microscopy.

### Immunofluoresence assay

After the required treatment, cells were fixed with ice-cold methanol for 10 min. Cells were washed with PBS for three times. Non-specific binding sites were blocked with 5% BSA for 30 min. These cells were incubated with the primary antibody (PCNA, 1:100, Abcam) overnight at 4°C, and then incubated with the secondary antibody conjugated with Alexa Fluor 594 (Invitrogen) for 3 h at room temperature, followed by incubation with 4′,6-Diamidino-2-phenylindole dihydrochloride (DAPI, Sigma) for 5 min.

Tissue slices were fixed in 4% paraformaldehyde in phosphate-buffered saline and embedded in paraffin. Immunohistochemistry was performed in paraffin sections (3 μm thick). The slices were incubated with the primary antibody (CD31, 1:100, BD Biosciences) overnight at 4°C, and then incubated with the secondary antibody conjugated with Alexa Fluor 594 (Invitrogen) for 3 h at room temperature, followed by incubation with DAPI for 10 min.

### Caspase-3 activity assay

Caspase-3 activity was detected using the caspase-3 activity kit (Beyotime, China). After the required treatment, cells were homogenized in reaction buffer (1% NP-40, 20 mM Tris-HCl (pH 7.5), 137 mM Nad and 10% glycerol) containing 10 ml caspase-3 substrate (Ac-DEVD-pNA) (2 mM). Lysates were incubated at 37°C for 1.5 h. Samples were detected using a plate reader (405 nm).

### Establishment of stable expression of CRNDE and CRNDE knockout cell line

Expression vectors encoding CRNDE or CRNDE shRNA were electroporated into HuH-6 cells. These cells were subsequently selected in blasticidin-containing medium for 7 days. Blasticidin-resistant colonies were examined for detecting the expression of CRNDE by qRT-PCRs. These cells were culture in blasticidin-containing medium for 4 weeks for cell selection.

### Enzyme-linked immunosorbent assay (ELISA)

VEGFA and Ang-2 ELISA kit (R&D Systems) was used to determine the activity of VEGFA and Ang-2 in cell culture supernatants and tissue lysates according to the manufacturer's recommendation. VEGFA, ESM-1, and CD34 levels in hepatoblastoma specimens and adjacent non-cancerous liver tissues were detected by ELISA assays. Briefly, the hepatoblastoma specimens and adjacent non-cancerous tissues were lysed using RIPA buffer and centrifuged. The collected supernatants were thawed and centrifuged to remove the particulate. The assay was performed according to the manufacturer's instructions. The optical density of each sample was determined using a microplate reader set to 450 nm with wavelength correction set to 570 nm. Data were expressed as mean concentration (pg/mL).

### Nude mouse xenograft model

Male C57BL/6 nude mice (6-weeks-old) were maintained in special pathogen-free (SPF) condition. The animal experiments were carried out according to the recommendations in the Guide for the Care and Use of Laboratory Animals of the National Institutes of Health. Mice were injected subcutaneously into the left flank with 1×10^7^ HuH-6 cells that have stable expression of scrambled shRNA or CRNDE shRNA, as 100-μL cell suspensions with an equal volume of Matrigel (BD Bioscience). Tumors were measured twice a week with a caliper, and tumor volumes (V) were calculated using the following formula, V= (L × W^2^) × 0.5. After 4-week tumor inoculation, mice were sacrificed and tumors were extracted [9].

### Statistical analysis

All data were expressed as means±S.E.M. unless indicated otherwise. Comparisons between groups were analyzed using Student's *t* test or Mann-Whitney *U*-test. *P*<0.05 was considered to be statistically significant. All results were reproduced in at least three independent experiments.
